# Efficient Assessment of Developmental, Surgical and Pathological Lymphangiogenesis Using a Lymphatic Reporter Mouse and Its Embryonic Stem Cells

**DOI:** 10.1371/journal.pone.0157126

**Published:** 2016-06-09

**Authors:** Mingu Hong, Eunson Jung, Sara Yang, Wonhyuek Jung, Young Jin Seong, Eunkyung Park, Athanasios Bramos, Kyu Eui Kim, Sunju Lee, George Daghlian, Jung In Seo, Inho Choi, In-Seon Choi, Chester J. Koh, Agnieszka Kobielak, Qi-Long Ying, Maxwell Johnson, Daniel Gardner, Alex K. Wong, Dongwon Choi, Young-Kwon Hong

**Affiliations:** 1 Division of Plastic and Reconstructive Surgery, Department of Surgery, Keck School of Medicine, University of Southern California, Los Angeles, California, United States of America; 2 Department of Biochemistry and Molecular Biology, Norris Comprehensive Cancer Center, Keck School of Medicine, University of Southern California, Los Angeles, California, United States of America; 3 Department of Pharmaceutical Engineering, Hoseo University, Asan, Republic of Korea; 4 Division of Pediatric Urology, Texas Children’s Hospital, Baylor College of Medicine, Houston, Texas, United States of America; 5 Centre of New Technologies, University of Warsaw, S. Banacha 2c, Room 2107, Warsaw, Poland; 6 Eli and Edythe Broad Center for Regenerative Medicine and Stem Cell Research, Department of Stem Cell Biology & Regenerative Medicine, Keck School of Medicine, University of Southern California, Los Angeles, California, United States of America; Feinberg Cardiovascular Research Institute, Northwestern University, UNITED STATES

## Abstract

Several lymphatic reporter mouse lines have recently been developed to significantly improve imaging of lymphatic vessels. Nonetheless, the usage of direct visualization of lymphatic vessels has not been fully explored and documented. Here, we characterized a new Prox1-tdTomato transgenic lymphatic reporter mouse line, and demonstrated how this animal tool enables the researchers to efficiently assess developmental, surgical and pathological lymphangiogenesis by direct visualization of lymphatic vessels. Moreover, we have derived embryonic stem cells from this reporter line, and successfully differentiated them into lymphatic vessels *in vivo*. In conclusion, these experimental tools and techniques will help advance lymphatic research.

## Introduction

Fluorescence-based visualization of vascular networks has profoundly contributed to the advancement of vascular research. The Tie2-GFP transgenic mouse model was the first vascular-specific fluorescent reporter mouse, allowing high-quality imaging of blood vessels. It is an extremely useful tool for endothelial cell isolation, as well as the evaluation of vascular morphogenesis, gene expression [1]. Similar efforts have been made to generate transgenic mouse lines to conveniently visualize lymphatic vessels. Initial attempts, however, relied on minimal promoters of lymphatic signature genes Vegfr-3 and podoplanin, and turned out to be unsuccessful due to insufficiency of these promoters in recapitulating the endogenous gene expression pattern [2, 3]. A transgenic mouse line harboring a reporter gene under the p105 gene promoter was later found to display lymphatic expression [4]. More recently, several groups have generated lymphatic reporter lines through various approaches by using large genomic fragments spanning lymphatic genes Prox1 and Vegfr-3 [5–8], by targeting a reporter into the genomic locus of lymphatic genes such as Lyve-1 and Prox1 [9, 10], or by combining a Prox1 promoter-regulated inducible Cre transgenic line with a reporter line (LSL-tdTomato) [11].

We have previously characterized a bacterial artificial chromosome (BAC)-based Prox1-EGFP transgenic mouse that enabled efficient visualization of lymphatic vessels [5]. This mouse, however, cannot be combined with other GFP-based reporter mice (e.g., Rosa26-EGFP reporter line for tissue specific Cre), or implanted with GFP-labeled cell lines (e.g., cancer or immune cells) due to overlapping usages of the EGFP gene. Therefore, we performed a series of analyses on the previously uncharacterized lymphatic reporter line Prox1-tdTomato. This line has been created by the Gene Expression Nervous System Atlas (GENSAT) BAC-transgenic mouse project using a modified mouse BAC (RP23-360I16) harboring the tdTomato reporter gene under the control of the Prox1 promoter [12]. Of note, the EGFP and CreER^T2^ genes have also been targeted into the Prox1 coding sequence in the same BAC clone to generate a lymphatic reporter [5] and a tamoxifen-inducible lymphatic-Cre mouse [13], respectively. These transgenic lines have been widely used as extremely useful animal models for the lymphatic research.

In this study, we characterize the reporter expression pattern in this new lymphatic reporter line Prox1-tdTomato, then evaluated the usefulness of direct visualization of the lymphatics in assessing surgical and pathological lymphangiogenesis in a range of experimental models. These models included lymph node dissection, tumor implantation, bladder inflammation and teratoma formation. We confirmed from all these models that Prox1-tdTomato mouse provided an excellent high-quality imaging of lymphatic vessels. Although several fluorescent lymphatic reporters have previously been generated [5–9, 11], an additional independent line may still be useful and provide more options to choose, mainly because the lymphatic reporter mice differ in chemical/fluorescent properties of the reporter proteins as well as chromosomal insertion sites, copy numbers and epigenetic status of the transgenes. Moreover, considering the fact that some transgenic mice tend to lose their transgene expression after several generations due to epigenetic silencing of the transgene, it will be important and beneficial to have multiple different reporter lines. Combined with previously generated lymphatic reporters, this lymphatic reporter mouse will be a valuable tool for imaging lymphatic growth, expansion and differentiation during physiological and pathological lymphangiogenesis.

## Materials and Methods

### Animal Usage

All animal-related studies have been approved by University of Southern California, Institutional Animal Care and Use Committee (IACUC) (PI: YK Hong). The Prox1-tdTomato mouse (*Tg(Prox1-tdTomato)TA76Gsat/Mmucd*) was developed by the Gene Expression Nervous System Atlas (GENSAT) Project and obtained from Mutant Mouse Regional Resource Centers (MMRRC). Prox1-EGFP mice were described previously [5]. Prox1-tdTomato and Prox1-EGFP mice in mixed backgrounds were used for this study. Athymic nude mice (*Foxn1*^*nu*^*/Foxn1*^*nu*^; the Jackson Laboratory) were intercrossed with Prox1-tdTomato, and outbred immunodeficient athymic Prox1-tdTomato mice were genotyped based on a protocol described in the Jackson Laboratory.

### Tumor Graft

One million GFP-labeled head and neck carcinoma cells (a gift from Agnieszka Kobielak, University of Warsaw) [14] were injected into the back skin of the athymic Prox1-tdTomato mice, which were anesthetized by isoflurane inhalant. The mice were then allowed to form tumors for 3 weeks. Then, tissues containing the tumors were harvested for stereoscopic imaging using a fluorescent stereomicroscope.

### Cyclophosphamide Treatment

Adult Prox1-tdTomato mice were i.p. injected with phosphate-buffered saline (PBS) or PBS containing cyclophosphamide (200 mg/Kg) into the peritoneum at days 1 and 4 (n = 3 per group). At day 9, the bladder was isolated and fixed in cold 4% paraformaldehyde (PFA) for 2 hours. After washing in PBS, the whole-mount images of the bladder were captured for lymphatic analyses.

### Axillary Lymphadenectomy

Axillary lymphadenectomy was performed as previously described [15]. Briefly, Prox1-EGFP or Prox1-tdTomato mouse were anesthetized using isoflurane inhalant. Axillary area was then sterilized by alcohol and betadine antiseptic solution and the skin was cut with a surgical scissors. Axillary lymph node was identified under a fluorescent light and dissected out. The skin was closed with surgical staples and 9-cis RA (0.08 mg/kg/day) was i.p. injected for the first 5 days after surgery. After 4 weeks, mice were euthanized and the dermal lymphatic vessels were visualized in the armpits of the mice for vascular analyses.

### Lymph Node Transplantation

For lymph node transfer, both Prox1-EGFP and Prox1-tdTomato mice were used as donor and recipient mice, respectively. The axillary areas were shaved and disinfected with alcohol. Both forepaws were injected with 10 μl of isosulfan blue (1%). Using a surgical operative microscope, all lymph nodes along with the perinodal fat as well as blue stained collecting lymphatic vessels were excised, to prevent possible lymphatic regrowth. Lymph nodes were harvested from Prox1-EGFP donor mice and immediately transferred under the pectoralis major muscle in the Prox1-tdTomato recipient mice. The incision was subsequently closed with interrupted nylon sutures. The mice were then i.p. injected with a vehicle (PBS, n > 3) or 9-cis RA (0.08 mg/kg/d, n > 3) for 4 weeks to enhance lymphangiogenic activity. After 4 weeks, the incision was re-opened and the transferred nodes were removed en bloc along with their adjacent fat. The harvested lymph nodes were fixed in 4% PFA overnight for further analyses. The experiment was repeated twice and more than 3 mice per group were used each experiment.

### Tail Lymphedema

Tail lymphedema was induced by following the previously established protocol [16]. Briefly, under a dissecting microscope, outbred Prox1-EGFP or Prox1-tdTomato mice were anesthetized by isoflurane inhalant and a circumferential 2-mm-wide piece of skin located about 1 cm-distal of the tail base was removed. Images of the surgery sites were captured in post-surgical days 1 and 15. At the end of the experiments (d15), mice were euthanized and a 1-cm long tail tissue that spans the circumcised wound was collected for visualization of new cutaneous lymphatic vessels that grew in the wounded area.

### Embryonic stem cells and teratoma formation

Isolation and culture of Prox1-tdTomato embryonic stem cells (ESCs) were performed as previous described [17]. One million ESCs were injected into the back skin of NOD-SCID IL2Rγnull mice. After 4 weeks, teratomas were harvested, fixed in 4% PFA overnight, and embedded for further immunohistochemistry analyses. Frozen sections were stained with Hematoxylin and Eosin (H&E) or stained for Cd31 and Lyve1 using rat anti-mouse Cd31 (BD Bioscience) and rabbit anti-mouse Lyve1 (Angiobio) antibodies. Cy5-conjugated anti-rat IgG and AlexaFluor 488-conjugated anti-rabbit IgG antibodies were used as secondary antibodies.

### Fluorescent Stereomicroscope Imaging

For whole mount tissue preparation, the ears were harvested from euthanized mice and the hairs were removed using a generic hair removal cream. The ears were briefly washed in PBS and fixed in 4% PFA at 4°C for 2 hours. Using fine forceps, the dorsal side- and ventral side ears were then separated and auricular cartilage was removed for a better visualization of the dermal lymphatic vessels. Prox1 staining was performed using anti-Prox1 antibody (R&D Systems). To take mouse headshots, anesthetized mice were shined by a UV fluorescent flashlight (NIGHTSEA, Bedford, MA) and the fluorescent images were captured using an iPhone camera. Other fluorescent stereoscopic images were taken using a Leica M165 stereomicroscope (Leica Microsystems).

## Results and Discussion

### Reporter Expression in the Prox1-tdTomato Embryos and Adults

**Fig 1:**
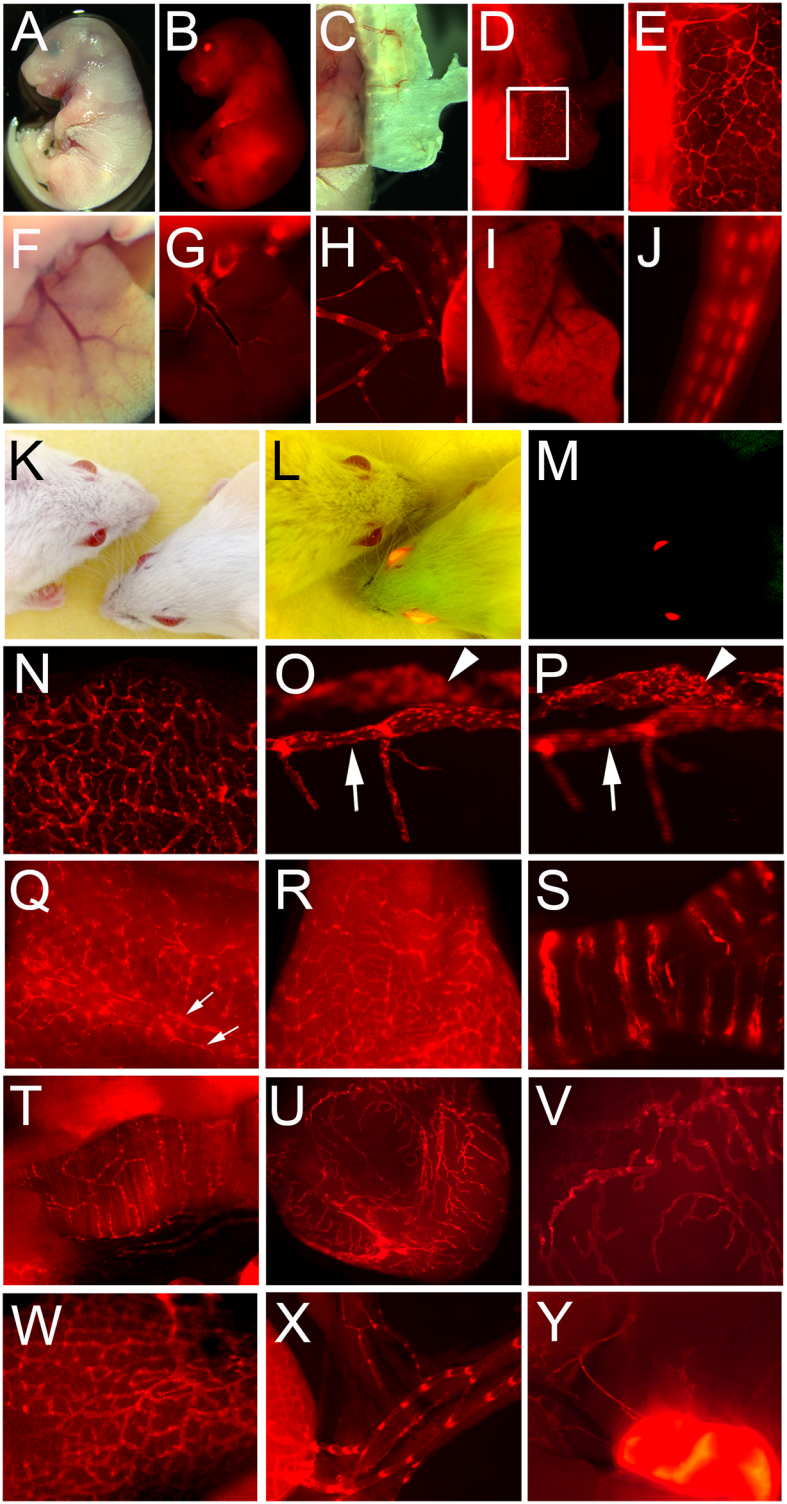
Characterization of the reporter expression in Prox1-tdTomato embryos and adults. (A-J) Embryonic tissues: bright field (A) and fluorescence (B) images of the Prox1-tdTomato embryo (E17.5). Distinct lymphatic networks shown in the embryonic skin (C-E). (E) Enlarged image of the boxed area in panel D. Lymphatic vessels in the embryonic liver (F,G) and mesentery membrane (H). Note that hepatocytes (I) and tail nerves (J) are also positive for tdTomato. (K-Y) Adult tissues: headshots of adult wild type and Prox1-tdTomato transgenic mice were taken under a bright light (K), bright and fluorescent light (L) and fluorescent light (M). Lymphatic vessels were easily detectable in various tissues such as the ear (N), eye (O,P), tail (Q), tongue (R), trachea (S), diaphragm muscle (T), bladder (U,V), intestine (W), mesentery (X) and lymph node (Y). Corneal limbal lymphatic (arrow) and Schlemm’s canal (arrowhead) of the eye were shown in two consecutive focal planes (O,P). Bilateral lymphatic collectors in the tail were marked with two arrows (Q).

We first investigated the expression pattern of the reporter gene during development of the Prox1-tdTomato mice. Embryos showed a very strong red signal in their lens (Fig 1A and 1B), consistent with the expression pattern of Prox1 [5]. In addition, developing lymphatic vessels were easily detectable in the embryonic skin, liver and mesentery (Fig 1C–1H). Other Prox1-expressing cells, including hepatocytes and neuronal cells, also displayed the tdTomato signal (Fig 1I and 1J).

We next characterized the reporter expression in adult mice. Like their embryos, Prox1-tdTomato adult mice could be easily distinguished by the strong reporter expression in their eyes under ultraviolet (UV) light (Fig 1K–1M). Importantly, there was substantial and consistent tdTomato expression in all lymphatic vessels. Capillary lymphatic vessels in the ears were sufficiently bright that hierarchical lymphatic networks could be readily visualized (Fig 1N). Similarly, branched limbal lymphatic vessels and neighboring unbranched Schlemm’s canal in the cornea were clearly detectable (Fig 1O and 1P). Bilateral collecting lymphatic vessels in the tail (Fig 1Q) and capillary lymphatic vessels in the tongue (Fig 1R) were also evident. Internal structures, including the trachea, diaphragm, bladder, intestine, mesentery and lymph nodes, also demonstrated well-organized lymphatic networks (Fig 1S–1Y). Taken together, these findings confirm that the new Prox1-tdTomato BAC transgenic mouse line displays a reporter expression pattern that is highly consistent with endogenous Prox1 expression.

### Direct Visualization of Lymphangiogenic Activity in Experimental Surgery Models

**Fig 2:**
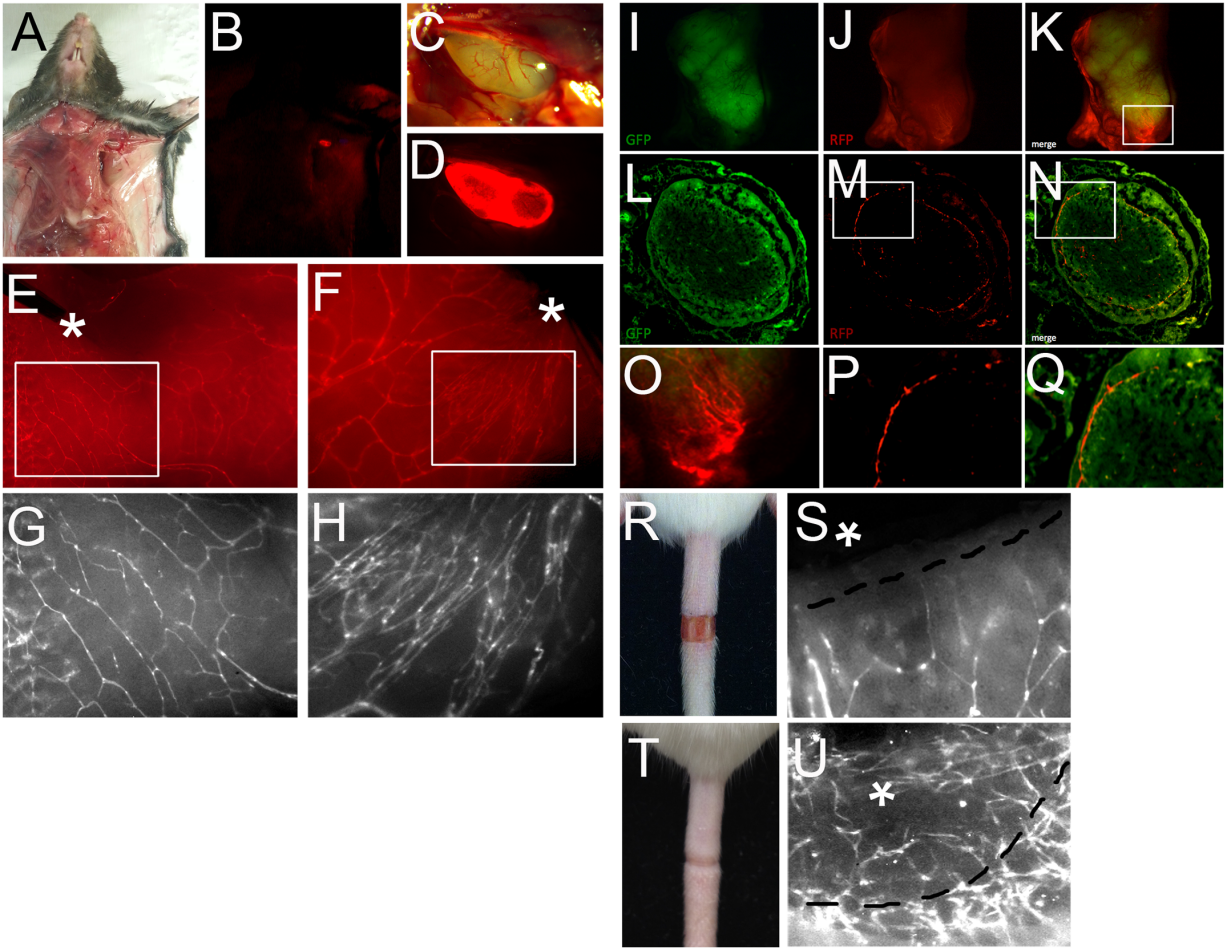
Surgical lymphangiogenesis models using the Prox1-tdTomato mouse. (A-H) Axillary lymphadenectomy model: Fluorescent light enables the researchers to easily locate the axillary lymph node (A-D). From a different set of mice, the left-side axillary lymph nodes were removed, while the right-side nodes were left intact. To enhance lymphatic growth in the surgical site, 9-cis RA was treated for the first 5 days after surgery. After 4 weeks, lymphatic vessels were visualized in the left *vs*. right side of the armpit of the mice with asterisks indicating the original locations of the lymph nodes (E,F). Images in the boxed areas (E, F) are shown in panels G and H. (I-Q) Lymph node transplantation model: the axillary lymph node in Prox1-tdTomato mice (recipient) was replaced with the matching lymph node from Prox1-EGFP mice (donor), followed by administration of 9-cis RA for 4 weeks. Panels O, P and Q are enlarged images of the boxed areas in panels K, M and N, respectively. (R-U) Tail lymphedema model: representative images of the circumcised tails at post-operational days 1 and 15 are shown (R,T). At both days, the circumcised tail skins were prepared inside-out to visualize dermal lymphatic vessels (S,U). Asterisks mark the wounded area and black dotted lines indicate the wound border. Note the numerous newly formed lymphatics invading into the healed area (S).

To demonstrate the values of this new reporter mouse in *in vivo* lymphangiogenesis assays, we performed three surgical procedures to visualize physiological lymphangiogenesis in association with wound healing. Unilateral axillary lymphadenectomy is a surgical procedure frequently performed in staging and treatment of breast cancer. Chronic lymphedema in the ipsilateral extremity is a common complication. This procedure has been employed to generate a murine limb lymphedema model to investigate the pathophysiology of lymphedema [18]. Measurements of lymphedema in this model, however, are often inconsistent and difficult to standardize, requiring additional criteria to grade the degree of lymphatic expansion and lymphedema. We hypothesized that the Prox1-tdTomato reporter line would significantly improve the quality of these measurements and performed unilateral axillary lymphadenectomy on the reporter mice to evaluate this relationship. Consistent with our previous findings, the axillary lymph nodes, which are otherwise difficult to locate, were readily identified under fluorescence light (Fig 2A–2D). We removed the left axillary lymph node, leaving the right axillary lymph node undisturbed, and closed the wound. To enhance lymphatic regrowth, the mice were treated with 9-cis retinoic acid (RA) for 5 days postoperatively [19]. After 4 weeks, we observed that numerous newly grown thin capillary lymphatic vessels were easily visualized in the left side. In contrast, no apparent lymphangiogenic activity was detected in the contralateral axilla (Fig 2E–2H). This demonstrates that the reporter-based direct visualization of lymphatic vessels could improve the evaluation of lymphatic expansion in surgical lymphangiogenesis models. This fluorescent-based direct visualization of lymphatic vessels allows keeping the tissue structure and morphology that are sometimes hard to maintain by the conventional immunostaining-based detection protocol.

Lymph node transplantation is used to treat patients with chronic lymphedema [20]. To evaluate the value of this new reporter line in associated experiments, we chose to perform lymph node transplantation in mice. We replaced the axillary lymph node in recipient Prox1-tdTomato mice with the matching lymph node isolated from donor Prox1-EGFP mice [5]. The mice were then treated with 9-cis RA for 4 weeks to enhance lymphangiogenic activity [19]. After 4 weeks, the transplanted lymph node and surrounding tissues were visualized to assess lymphangiogenic activity. Donor tissue expressed green fluorescence, while host tissue was red under fluorescent light. Interestingly, new lymphatic capillaries from the recipient tissue were found to infiltrate into the cortex of the implanted lymph node (Fig 2I–2Q). This suggests that host-derived lymphangiogenic activity may contribute to the anastomosis of donor and recipient lymphatics after lymph node transplantation. Further, our model could potentially be useful to study this relationship in future experiments.

Finally, we used a mouse tail lymphedema model to demonstrate how direct visualization of lymphatics could help *in vivo* lymphangiogenic assays. The mouse tail lymphedema model is widely used to study secondary lymphedema [16, 19]. In this model, a 2-mm-wide circumferential wound is made in the tail skin at approximately 1-cm from the tail base, which largely blocks dermal lymphatic-mediated fluid transport and causes fluid accumulation and swelling at the distal side of the wound within a few days [16, 19]. Although the degree of tail swelling is a good indicator of lymphedema progression, the difficulty of quantifying newly formed lymphatics is a primary limitation of this model. We asked whether Prox1-tdTomato mice could enable an efficient visualization of any newly formed lymphatics in the tail, and thus applied this lymphedema model to Prox1-tdTomato mice. After wound healing, a 1-cm long fragment of tail containing the wound site was harvested, and a longitudinal cut was made to remove tailbones prior to imaging. Evaluation of the subcutaneous side of the wounded tissue under UV light clearly demonstrated numerous fine lymphatic capillaries growing across the wound boundary into the healed area (Fig 2R–2U). Together, our studies demonstrate the advantages of using this new reporter line in various surgical/wound-related *in vivo* lymphangiogenesis assays.

### Convenient Detection of Lymphatic Vessels in Pathological Lymphangiogenesis Models using Prox1-tdTomato Mice

**Fig 3:**
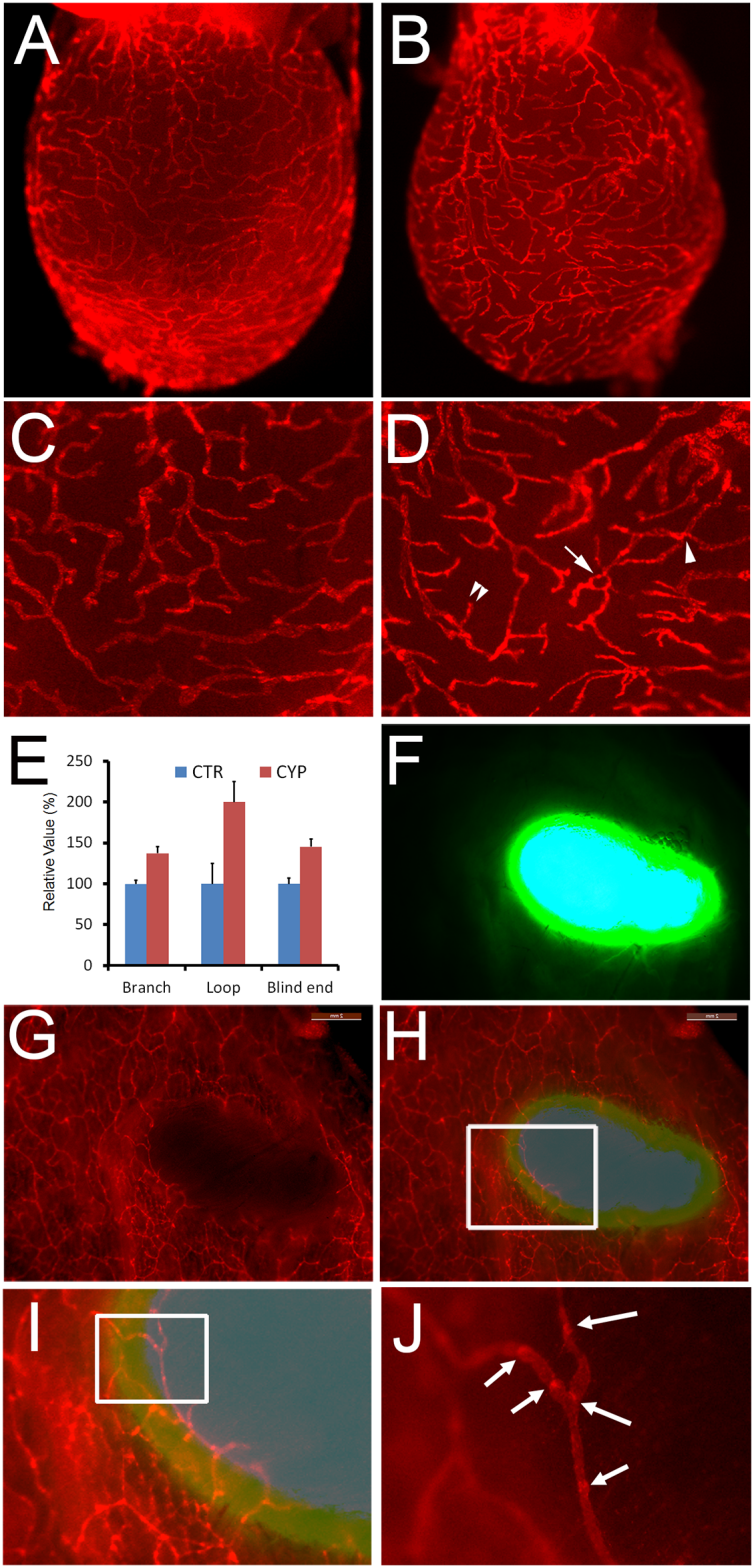
Pathological lymphangiogenesis models using Prox1-tdTomato mouse. (A-E) Bladder lymphangiogenesis model: Adult Prox1-tdTomato mice were i.p. injected with phosphate-buffered saline (A,C) or cyclophosphamide (B,D) at days 1 and 4. At day 9, lymphatic vessels in their bladders were visualized. Morphometric analyses revealed a significantly increase in the number of branch (marked with arrowhead), loop (arrow) and blind ends (double arrowhead) of lymphatic vessels in the CYP-treated bladder compared to the PBS-treated control (CTR) bladder (E). (F-J) Tumor implantation model: GFP-labeled tumor cells were implanted into the skin of immunodeficient athymic Prox1-tdTomato mouse, generated by crossing athymic nude mice and Prox1-tdTomato mice. Images of the GFP-labeled tumor mass (F), dermal lymphatic in the surrounding tissues (G) and their merged image (H) are shown. (I) and (J) are enlarged images of the boxed areas in panel (H) and (I), respectively. Arrows mark luminal valves.

In addition to their value in physiological lymphangiogenesis models, Prox1-tdTomato mice proved useful for the study of pathological lymphangiogenesis. First, we found that the ability to directly visualize lymphatic vessels in the bladder of the reporter line could be an excellent assay system to evaluate the lymphangiogenic potency of certain chemical compounds or drugs. As a proof of concept, we evaluated the effect of cyclophosphamide on *in vivo* lymphangiogenesis. Cyclophosphamide, a DNA alkylating anti-cancer drug, is commonly employed in chemotherapy for a wide range of cancers. Cyclophosphamide has severe adverse effects, most notably urinary bladder toxicity with inflammation and cancer [21]. Cyclophosphamide was injected into Prox1-tdTomato mice two times (Days 1 and 4) over a week period and then the bladder was harvested for lymphatic analysis under a fluorescent microscope. Indeed, lymphatic vessels in the bladder were easily visualized and their morphometric analyses could be conveniently carried out largely due to its membranous structure (Fig 3A–3D). Importantly, cyclophosphamide activated bladder lymphangiogenesis by increasing the number of branch, loop and blind end sprouts of lymphatic vessels (Fig 3E). Of note, we did not find any significant dermal lymphangiogenic activities in the ears (data not shown), implying that the effect of cyclophosphamide seems to be limited to the visceral/urinary organs. As the bladder can be stretchable, it was important to empty the urine to normalize the bladder area before histological treatment and subsequent vascular analyses.

As a second pathological lymphangiogenesis model, we studied the morphological characteristics of peri-tumoral lymphatic capillaries in Prox1-tdTomato mice. For this purpose, we intercrossed Prox1-tdTomato mice with athymic nude mice (*Foxn1*^*nu*^*/Foxn1*^*nu*^) and generated an immunocompromised athymic lymphatic reporter line. We then implanted EGFP-labeled human head and neck squamous cell carcinoma (HNSCC) cells into the back skin of the athymic Prox1-tdTomato mice. After 3 weeks, the skin was harvested and whole-mount images of the tumor/lymphatic interface were captured. Indeed, recipient lymphatic vessels were conveniently and clearly seen invading the stroma of the EGFP-labeled tumor (Fig 3F–3I). Interestingly, these invading lymphatic capillaries were equipped with luminal valves (arrows in Fig 3J), which were characterized by their high level of Prox1 (tdTomato) expression [22]. Taken together, our study shows that the Prox1-tdTomato mouse provides a significant imaging advantage for pathological lymphangiogenesis models.

### Derivation and Usage of Prox1-tdTomato Embryonic Stem Cells

**Fig 4:**
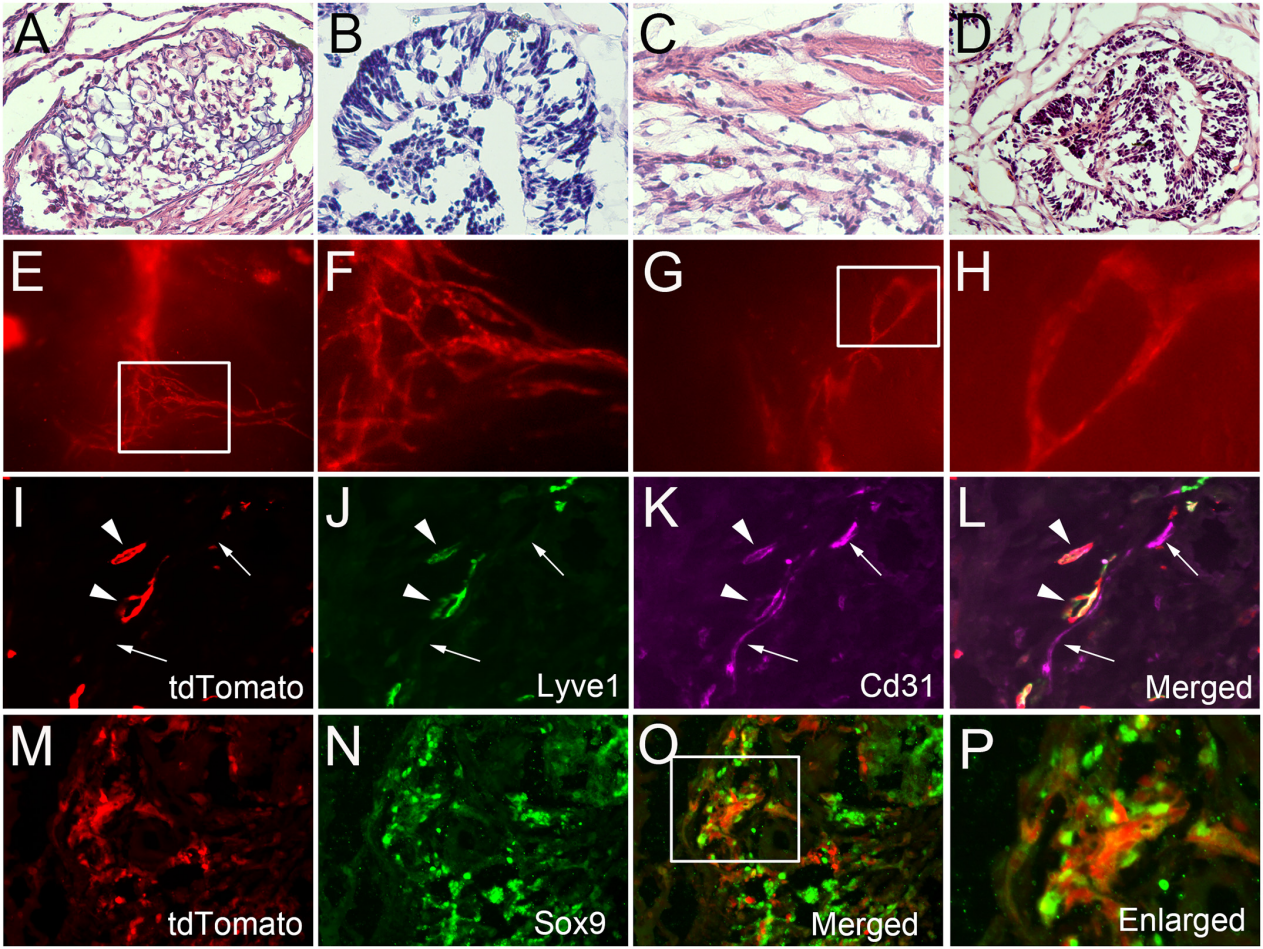
Lymphatic differentiation of embryonic stem cells derived from Prox1-tdTomato mice. (A-D) H&E stain showing that lymphatic reporter ES cells could form teratoma and differentiate into various cell types such as cartilage (A), gut epithelial cells (B), muscle-like (C) and neuroectodermal tissues (D). (E-H) Whole-mount images of tdTomato-positive lymphatic vessels formed on the surface of teratomas. (F) and (H) are enlarged images of the boxed areas in panel (E) and (G), respectively. (I-L) Immunofluorescent stains for Lyve1 and Cd31 on a frozen section of teratoma. Arrowheads mark Cd31^+^/tdTomato^+^/Lyve1^+^ lymphatic vessels, whereas arrows indicate Cd31^+^/tdTomato^-^/Lyve1^-^ blood vessels. (M-P) Co-expression of tdTomato protein and Sox9 in the putative hepatic progenitor cells.

Lymphatic endothelial cell differentiation involves a series of complex molecular and cellular events that are not fully understood. One major limitation in the study of lymphatic cell fate specification is the inability to directly detect lymphatic endothelial cell differentiation *in situ*. Therefore, we hypothesized that ESCs from Prox1-tdTomato mice would be a useful resource to detect lymphatic differentiation and set out to isolate and culture Prox1-tdTomato ESCs. For this purpose, we isolated E3.5 blastocysts from pregnant female mice and derived multiple ESC lines. We assessed the pluripotency of two of these lines by injecting them into the skin of immunodeficient NOD-SCID IL2Rγnull mice and observed for teratoma formation. After 4 weeks, tissues harboring teratomas were harvested and subjected to histological analyses. H&E stains confirmed that the Prox1-tdTomato ESCs formed teratomas with characteristic tissues of all three germ layers (Fig 4A–4D). More importantly, we could easily detect sprouting lymphatic vessels and lymphatic loops on the surface of these teratomas under UV light (Fig 4E–4H). Subsequently, frozen cross sections were prepared from the teratomas and subjected to immunofluorescent analyses (Fig 4I–4L). These analyses revealed numerous tdTomato-positive intratumoral lymphatic vessels that also expressed the lymphatic-signature gene Lyve1 and pan-endothelial marker Cd31, indicating that the Prox1-tdTomato ESCs can differentiate into lymphatic endothelial cells and form lymphatic vascular structures. In addition, we asked if the tdTomato-positive cells in the non-vascular structures express other Prox1-expressing cells markers. Previous studies show that hepatic progenitor cells express Prox1 [23, 24]. Consistent with these studies, we found that some prominent tdTomato-expressing cells in the teratoma strongly express Sox9 (Fig 4M–4P), suggesting that they are likely to be progenitor cells that could form the primitive ductal structures. Taken together, we have successfully derived pluripotent ESC lines from Prox1-tdTomato mice and these novel lymphatic reporter ESCs can differentiate into endothelial cells that form vessels expressing LYVE1. These ES cells could be a valuable resource for direct monitoring of lymphatic differentiation *in situ*.

### Further confirmation of the expression of the tdTomato reporter protein in LECs

**Fig 5:**
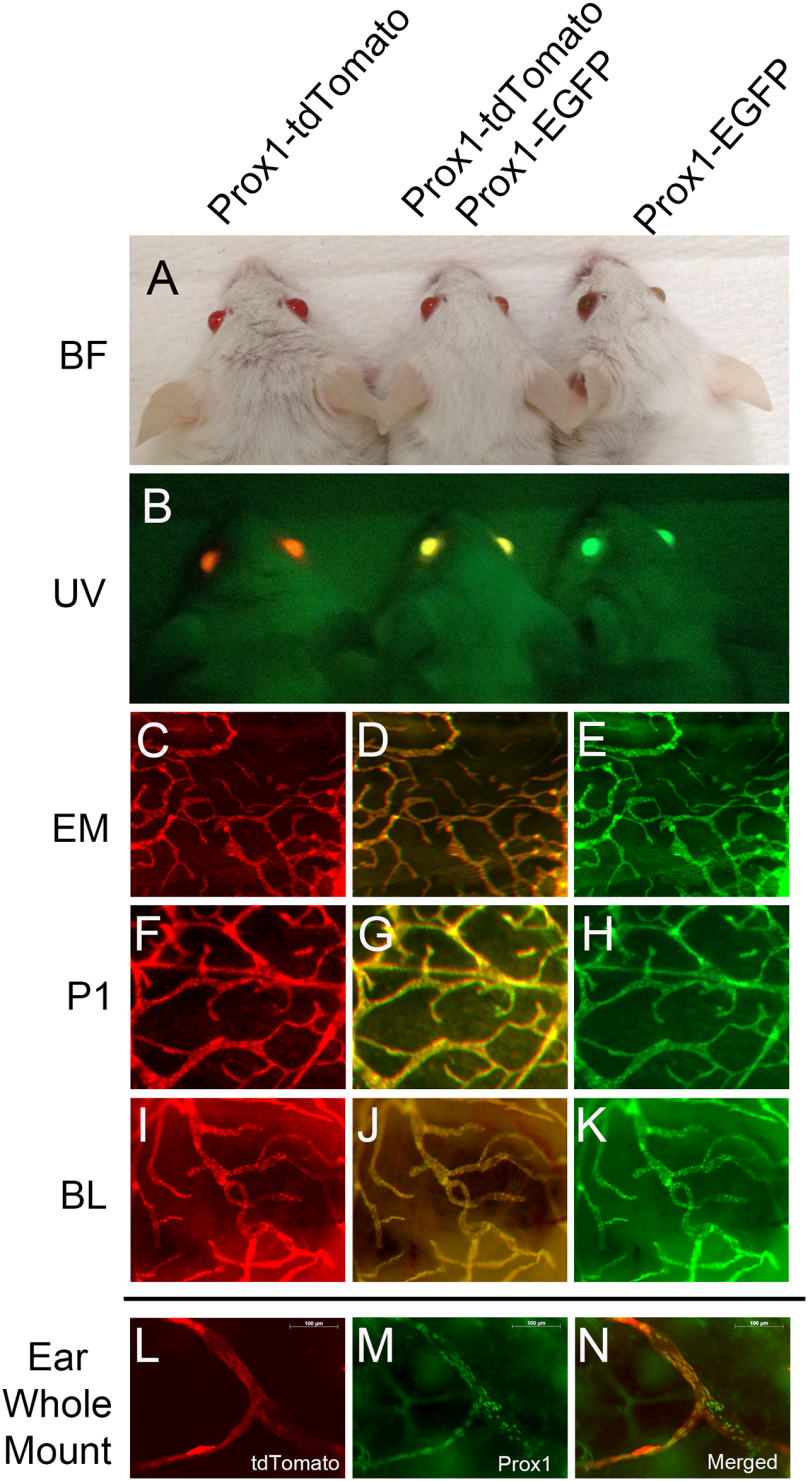
Confirmation of lymphatic-specific expression of tdTomato reporter protein. (A,B) Bright field (BF) and fluorescent (UV) images of the head of Prox1-tdTomato, Prox1-tdTomato/Prox1-EGFP and Prox1-EGFP lymphatic-specific reporter mice. (C-K) Expressions of tdTomato and/or EGFP proteins were visualized in the skin of embryos (EM, C-E) and new pups (P1, F-H), or in the bladder of adult (BL, I-J) of each genotype. (L-N) Prox1 whole-mount staining of the ear of tdTomato adult mouse.

In order to investigate whether this new Prox1-tdTomato reporter mice display any positional, epigenetic and/or copy number effects that often result in improper mis-expressions or failed-expressions of the reporter, we inter-crossed this reporter with the Prox1-EGFP lymphatic reporter mice to generate double transgenic mice (Prox1-tdTomato/Prox1-EGFP) and performed comparative analyses of the expression of both reporter proteins. Indeed, this double tdTomato/EGFP reporter mouse showed a characteristic yellow color in their eye lens (Fig 5A and 5B). We obtained embryos, new pups and adults that carry these two reporters and confirmed that all tested lymphatic vessels in various organs faithfully expressed both reporter proteins (Fig 5C–5K). We could not find any lymphatic vessels that express only single reporter protein. Moreover, we performed Prox1 whole-mount staining against dermal lymphatic vessels in the ear of Prox1-tdTomato mice. As expected, tdTomato-positive lymphatic vessels clearly express Prox1 (Fig 5L–5N). Taken together, these data indicate that Prox1-tdTomato and Prox1-EGFP mice, which were independently created, do not display any discrepancies and differences in their lymphatic-specific reporter expression patterns.

## Conclusions

In this report, we characterized a new transgenic lymphatic reporter Prox1-tdTomato mouse. We confirmed that the Prox1-tdTomato mouse enables a direct visualization of lymphatic vessels. Employing various surgical and pathological lymphangiogenesis models, we demonstrated the usefulness and value of this new lymphatic reporter mouse in detecting *in vivo* lymphangiogenic activities. We proved that direct visualization of lymphatics could significantly corroborate the functional readouts of the existing lymphangiogenesis models. Moreover, we have successfully derived pluripotent ESC lines from the Prox1-tdTomato mouse, which will enable a convenient detection of lymphatic differentiation *in situ*. These new ESCs could be extremely useful for the real time, live imaging of lymphatic endothelial differentiation, which will enable us to better understand the key molecular and cellular events during the lymphatic development process. In conclusion, our newly developed experimental resource will significantly help advance lymphatic research.
